# A Pilot Study of Serum MicroRNAs Panel as Potential Biomarkers for Diagnosis of Nonalcoholic Fatty Liver Disease

**DOI:** 10.1371/journal.pone.0105192

**Published:** 2014-08-20

**Authors:** Youwen Tan, Guohong Ge, Tengli Pan, Danfeng Wen, Jianhe Gan

**Affiliations:** 1 Department of Infectious Diseases, The First Affiliated Hospital of Soochow University, Suzhou, China; 2 Department of Hepatosis, The Third Hospital of Zhenjiang Affiliated Jiangsu University, Zhenjiang, China; University of Hong Kong, Hong Kong

## Abstract

**Background:**

The invasive nature of liver biopsy makes the histopathological diagnosis of non-alcoholic fatty liver disease (NAFLD) difficult and its diagnostic performance unsatisfactory. The present study aimed to identify a serum microRNA (miRNA) expression profile that could serve as a novel diagnostic biomarker for NAFLD.

**Methods:**

Serum miRNA expression was investigated using three cohorts comprising 465 participants (healthy controls and NAFLD patients) recruited between August 2010 and June 2013. miRNA expression was initially screened by Illumina sequencing using serum samples pooled from 20 patients and 20 controls. Quantitative reverse transcriptase polymerase chain reaction assay was then used to evaluate the expression of selected miRNAs. A logistic regression model was constructed using a training cohort (n = 242) and validated using another cohort (n = 183). The area under the receiver operating characteristic curve (AUC) was used to evaluate diagnostic accuracy.

**Results:**

We identified an miRNA panel (hsa-miR-122-5p, hsa-miR-1290, hsa-miR-27b-3p, and hsa-miR-192-5p) with a high diagnostic accuracy for NAFLD. The satisfactory diagnostic performance of the miRNA panel remained regardless of the NAFLD activity score (NAS) status. There was significant difference between the AUC values of the miRNA panel and those of ALT (AUC = 0.786, 95% CI = 0.717–0.855; *P* = 0.142) and FIB-4 (AUC = 0.795, 95% CI = 0.730–0.860; sensitivity = 69.9%, specificity = 83.7%.

**Conclusion:**

We identified a serum microRNA panel with considerable clinical value in NAFLD diagnosis. The results indicate that the miRNA panel is a more sensitive and specific biomarker for NAFLD than ALT and FIB-4.

## Introduction

Non-alcoholic fatty liver disease (NAFLD) is an acquired metabolic stress-induced liver disease associated with insulin resistance (IR) and genetic susceptibility. It has histological similarities with alcoholic liver disease (ALD) in the absence of substantial alcohol consumption or other causes of liver disease. The spectrum of NAFLD ranges from simple steatosis to non-alcoholic steatohepatitis (NASH) and eventually, cirrhosis and hepatocellular carcinoma. Currently, NAFLD is one of the important public health concerns worldwide, and more so in China [1]. A liver biopsy is the gold standard for the diagnosis of NAFLD. However, this procedure has well-known limitations (invasiveness and sampling variability) and thus cannot be proposed for all patients, given the high prevalence of NAFLD worldwide [2].

MicroRNAs (miRNAs) are an emerging class of highly conserved, non-coding small RNAs that regulate gene expression at the post-transcriptional level. It is now clear that miRNAs can potentially regulate every aspect of cellular activity, including differentiation and development, metabolism, proliferation, apoptotic cell death, viral infection, and tumorigenesis [3]. Recent studies provide clear evidence that miRNAs are abundant in the liver and modulate a diverse spectrum of liver functions [4]. Deregulation of miRNA expression may be a key pathogenic factor in many liver diseases including viral hepatitis, hepatocellular cancer, and polycystic liver disease. A clearer understanding of the mechanisms involved in miRNA deregulation would offer new diagnostic and therapeutic strategies to treat liver diseases. Circulating miRNAs, which are extremely stable and protected from RNAase-mediated degradation in body fluids, have emerged as candidate biomarkers for many diseases [5], [6], [7]. The use of miRNAs as noninvasive biomarkers is of particular interest in liver diseases [8], [9], [10].

Since the initial study by Cheung et al showing differential expression of 46 (23 up-regulated and 23 down-regulated) hepatic miRNAs in patients with NASH and metabolic syndrome compared to subjects with normal liver histology [11], a number of additional studies have been conducted, mostly in animal models of NAFLD [12], [13], [14].

Our study investigated miRNA expression profiles with independent validation in a large cohort of participants, in order to identify a panel of miRNAs for the diagnosis of NAFLD. The cohort included healthy individuals and NAFLD patients.

## Materials and Methods

### Ethics statement

The study was approved by the Medical Ethics Committee of The First Affiliated Hospital of Soochow University and The Third Hospital Affiliated to Jiangsu University (No. 2012076 and No. 282), and written informed consent was obtained from each patient prior to participation. The study was conducted in accordance with the Declaration of Helsinki.

### Study design, patients, and healthy controls

**Figure 1:**
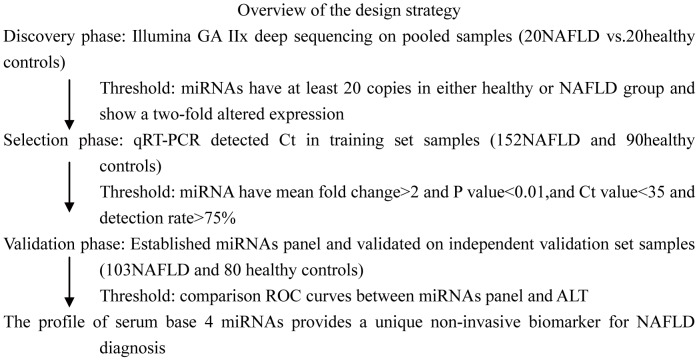
A flow chart of the experimental design.

A multistage, case-control study was designed to identify a serum miRNA profile as a surrogate marker for NAFLD (Fig. 1). A total of 275 NAFLD patients and 190 healthy controls were enrolled in our study. In the discovery biomarker screening stage, NAFLD serum samples pooled from 20 healthy control donors and 20 NAFLD patients treated at The First Affiliated Hospital of Soochow University were subjected to Illumina GA IIx deep sequencing to identify the miRNAs that were significantly differentially expressed. Subsequently, sequential validation was performed using a hydrolysis probe-based qRT-PCR assay to refine the number of serum miRNAs as an NAFLD signature. In the biomarker selection stage, 152 NAFLD serum samples and 90 controls (from The First Affiliated Hospital of Soochow University and The Third Hospital Affiliated Jiangsu University) formed a training set, whereas an additional 103 NAFLD serum samples and 80 normal subjects (from The Third Hospital of Zhenjiang Affiliated Jiangsu University) formed an independent validation set. All patients were diagnosed with NAFLD between August 2010 and June 2013, and blood samples were collected prior to any therapeutic procedure. After 8 h fasting, abdominal ultrasound was performed for all the enrolled patients using FFsonic UF-4100 (Fukuda Denshi, Tokyo, Japan). The liver echo pattern was graded according to the classification by Mottin et al [15]. Patients with disorders such as drug-induced liver disease, alcoholic liver disease, viral hepatitis, schistosomiasis, autoimmune hepatitis, primary biliary cirrhosis, sclerosing cholangitis, α_1_-antitrypsin deficiency, hemochromatosis, Wilson's disease, and biliary obstruction were excluded from the study. Those who had recently undergone gastrointestinal surgery, pregnant women, patients suffering from any malignancy, or those under any kind of medication were also excluded.

It is necessary to perform liver biopsy for patients diagnosed with NAFLD via ultrasound. The diagnosis of NAFLD requires the presence of the following features [1]: (i) the histological findings of liver biopsy are consistent with the pathological diagnostic criteria of fatty liver disease; the NAFLD activity score (NAS) be assessed routinely to make a pathological diagnosis according to Kleiner et al's NAS scoring system [16], according to which patients with NAS<3 were considered not having NASH; patients with scores ≥5 were diagnosed as having NASH; and those with scores between 3 and 5 were diagnosed as probably having NASH (ii) there is no history of alcohol consumption or ethanol intake per week is <140 g in men (70 g in women) in the 12 months preceding the study.

The demographics and clinical features of the patients are listed in Table 1. The NAS features of NAFLD are shown in Table S1. Healthy control subjects were recruited from a large pool of individuals seeking a routine health check-up at the Healthy Physical Examination Centre of The First Affiliated Hospital of Soochow University who showed no evidence of NAFLD by abdominal ultrasound. Patients with other disorders such as drug-induced liver disease, alcoholic liver disease, viral hepatitis, schistosomiasis, autoimmune hepatitis, primary biliary cirrhosis, sclerosing cholangitis, α1-antitrypsin deficiency, hemochromatosis, Wilson's disease, and biliary obstruction were excluded. The healthy controls were also required to have normal ALT level (ALT<40 IU/ml) and no history of coronary heart disease, hypertension, valvular disease, any arrhythmia or systemic disease for inclusion in the study. The controls and patients were matched based on age, gender, and ethnicity.

**Table 1:** Demographic and clinical features of NAFLD patients and healthy controls in the screening set.

	screening set				
Variables	NAFLDs (n = 20)		Controls (n = 20)		*p*-value
	N0.	%	N0.	%	
Average age(years)	40.25±7.57		39.1±6.83		*p* = 0.617^a^
Sex					
Male	15	75	16	80	*p* = 0.705^b^
Female	5	25	4	20	
BMI^1^	25.19±1.74		22.49±1.36		*p* = 0.000^a^
Smoking status					
Ever	3	15	2	10	*p* = 0.865^b^
Current	5	25	6	30	
Never	12	60	12	60	
Alcohol consumption					
Occasional**^2^**	15	75	12	60	*p* = 0.311^b^
Never	5	25	8	40	
NAS**^3^**					
<3	6	30			
≥3∼5	10	50			
≥5	4	20			
ALT(U/L)	52.55±44.02		27.35±7.48		*p* = 0.016^a^
AST(U/L)	54.1±43.78		27.5±4.81		*p* = 0.01^a^
Platelets(10^9^/L)	131.5±21.95		153.12±22.65		*p* = 0.004^a^

1 BMI: Body mass index, **^2^**Occasional: the ethanol intake per week was less than 140 g in men (70 g in women) in the past 12 months.**^3^**NAS: the NAFLD activity score. ^a^Independent samples-t test. ^b^Pearson Chi-Square.

### RNA isolation and library preparation

About 5 mL of venous blood was collected from each participant. The whole blood was separated into serum and cellular fractions by centrifugation at 4,000 rpm for 10 min, followed by 5-min centrifugation at 13,000 rpm for complete removal of cell debris. The supernatant serum was stored at −80°C until analysis. Total RNA was isolated using LCS TRK1001 miRNeasy kit (LC Sciences, Hangzhou, China). The libraries were constructed from total RNA using the Illumina Truseq Small RNA Sample Preparation Kit (Illumina, San Diego, CA, USA) according to the manufacturer's protocol. Briefly, RNA 3′ (P-UCGUAUGCCGUCUUCUGCUUG-UidT) and 5′ (GUUCAGAGUU CUACAGUCCGACGAUC) adapters were ligated to target miRNAs in two separate steps. Reverse transcription reaction was applied to the ligation products to create single stranded cDNA. The cDNA was amplified by PCR using a common primer and a primer containing the index sequence (CAAGCAGAAGACGGCATACGA). The quantity and purity of total RNAs were monitored using a NanoDrop ND-1000 spectrophotometer (NanoDrop Inc, Wilmington, DE, USA) at a 260/280 ratio >2.0. The integrity of total RNAs was analyzed using an Agilent 2100 Bioanalyzer system and RNA 6000 Nano LabChip Kit (Agilent Tech, Santa Clara, CA, USA) with RNA integrity number >8.0. Finally, Illumina sequencing technology was employed to sequence these prepared samples.

### Illumina sequencing and data analysis

The raw sequences were processed using the Illumina pipeline program. After masking of adaptor sequences and removal of contaminated reads, the clean reads were filtered for miRNA prediction with the software package ACGT101-miR-v3.5 (LC Sciences, Houston, Texas, USA) and subsequently analyzed according to (http://www.lc-bio.com/products/available_arrays.asp?id=181). Secondary structure prediction of individual miRNAs was performed by Mfold software (Version 2.38; http://mfold.rna.albany.edu/?q=mfold/RNA-Folding-Form) using the default folding conditions. The raw dates were reduced to cleaned sequences by removal of the following sequences: (1) 3ADT&length filter: reads were removed due to 3ADT not being found, and reads with length <18 and >26 were removed. (2) Junk reads: Junk: ≥2N, ≥7A, ≥8C, ≥6G, ≥7T, ≥10Dimer, ≥6Trimer, or ≥5Tetramer. (3) Rfam: Collection of many common non-coding RNA families except miRNAs (http://rfam.janelia.org). (4) Repeats: Prototypic sequences representing repetitive DNA from different eukaryotic species (http://www.girinst.org/repbase). (5) Notes: There was overlap in mapping of reads with mRNA, rRNA, tRNA, snRNA, snoRNA, and repeats. (6) mRNA Database: (http://www.ncbi.nlm.nih.gov/). The clean sequence reads were mapped with miRBase 20.0, allowing a mismatch of one or two nucleotide bases. More detailed description of the computational pipeline employed for data handling is reported in a flow-chart outline of study procedures (Figure S1). All data were transformed to log base 2. Differences between the samples were calculated using chi-square and fisher's exact test. Only miRNAs with fold difference >2.0 and *P*<0.05 were considered statistically significant.

### qRT-PCR validation study and data analysis

qRT-PCR-based relative quantification of miRNAs (300 µL of serum from each participant) was performed with SYBR Premix Ex Taq (TaKaLa) according to the manufacturer's instructions using a Rotor-Gene 3000 Real-time PCR machine (Corbett Life Science, Sydney, Australia). The RT primers and realtime PCR primers were designed as described [17]. Briefly, 1 µg of total RNA was reverse transcribed under the following conditions:16°C for 15 min, 42°C for 60 min, and 85°C for 5 min. The 20 µl PCR included 1 µl RT product and 1 µl EvaGreen dye (Biotium, Hayward, CA). The conditions for the PCR reaction were as follows: 95°C for 5 min followed by 40 cycles of 95°C for 15 s and 60°C for 1 min using an ABI PRISM 7300 thermal cycler. All reactions were run in triplicate. The threshold cycle (Ct) is defined as the fractional cycle number at which the fluorescencepasses the fixed threshold.According to the results obtained, miRNA-24 has been reported to be consistently present in human serum [18], [19]. Moreover, our previous experience is that miRNA-24 maintains a stable expression, and that the level of miRNA-24 served as an internal control in serum miRNA relative quantitative analysis. The specificity of each PCR product was validated by melting curve analysis at the end of PCR cycles. All samples were analyzed in triplicate, and the cycle thresholdvalue was defined as the number of cycles required for the fluorescent signal to reach the threshold. The relative expression levels of miRNAs in serum were calculated using the formula 2^−ΔΔCt^ where ΔΔCt = [Ct (target, test)−Ct (ref, test)]−[Ct (target, calibrator)−Ct (ref, calibrator)] [20]. All primers used were obtained from Invitrogen company (Shanghai, China).

### Statistical analysis

All Illumina sequencing data were transformed to log base 2. Differences between the samples were calculated using chi-square and fisher's exact test. Only miRNAs with fold difference >2.0 and *P*<0.05 were considered statistically significant. Data were presented as median ± SD. The data of demographic and clinical features of the NAFLD patients and healthy controls were analyzed using the Statistical Package for the Social Sciences (SPSS) version 21.0 software (SPSS Inc, Chicago, IL, USA). For the data 2^−ΔΔCt^ of miRNAs obtained by qRT-PCR, Mann-Whitney unpaired test was used to compare between NAFLD patients and controls. A stepwise logistic regression model was used to select diagnostic miRNA markers based on the training dataset. The predicted probability of being diagnosed with NAFLD was used as a surrogate marker to construct the receiver operating characteristic (ROC) curve. Area under the ROC curve (AUC) was used as an accuracy index for evaluating the diagnostic performance of the selected miRNA panel. The ROC and regression analysis was performed using the software 21 MedCalc (Version 10.4.7.0; MedCalc, Mariakerke, Belgium). All *P*-values were two-sided.

## Results

### Description and clinical features of patients

All 275 patients enrolled in the present study were clinically and pathologically diagnosed with NAFLD. As shown in Table 1–3, there were no significant differences in the distribution of smoking, alcohol consumption, age, and gender between NAFLD patients and normal subjects. However, the BMI, ALT, AST and platelets levels of NAFLD patients were significantly different from those of the normal controls.

**Table 2:** Demographic and clinical features of NAFLD patients and healthy controls in the training set.

	training set				
Variables	NAFLDs(n = 152)		Controls(n = 90)		*p*-value
	N0.	%	N0.	%	
Average age(years)	39.46±6.86		39.91±7.23		*p* = 0.629^a^
Sex					
Male	123	80.9	72	80	*p* = 0.861^b^
Female	29	19.1	18	20	
BMI^1^	24.74±1.92		22.74±1.49		*p* = 0.000^a^
Smoking status					
Ever	21	13.8	14	15.6	*p* = 0.071^b^
Current	32	21.1	32	33.3	
Never	99	65.1	44	51.1	
Alcohol consumption					
Occasional^2^	111	73	52	61.1	*p* = 0.054^b^
Never	41	27	38	38.9	
NAS^3^					
<3	32	30.3			
≥3∼5	72	47.4			
≥5	34	22.4			
ALT(U/L)	44.52±21.13		30.96±6.27		*p* = 0.000^a^
AST(U/L)	48.75±30.00		30.13±6.26		*p* = 0.000^a^
Platelets(10^9^/L)	135.42±21.7		154.3±24.6		*p* = 0.000^a^

1 BMI: Body mass index, ^2^Occasional: the ethanol intake per week was less than 140 g in men (70 g in women) in the past 12 months.^3^NAS: the NAFLD activity score. ^a^Independent samples-t test. ^b^ Pearson Chi-Square.

**Table 3:** Demographic and clinical features of the NAFLD patients and healthy controls in the validation set.

	validation set				
Variables	NAFLDs(n = 103)		Controls(n = 80)		*p*-value
	N0.	%	N0.	%	
Average age(years)	42.37±6.71		40.68±7.72		*p* = 0.118^a^
Sex					
Male	77	74.8	59	73.8	*p* = 0.877^b^
Female	26	26.2	21	26.2	
BMI^1^	24.89±1.98		22.26±1.52		*p* = 0.000^a^
Smoking status					
Ever	15	14.6	14	17.5	*p* = 0.734^b^
Current	32	31.1	27	33.8	
Never	56	54.4	39	48.8	
Alcohol consumption					
Occasional^2^	73	70.9	54	67.5	*p* = 0.623^b^
Never	30	29.1	26	32.5	
NAS^3^					
<3	22	21.4			*p* = 0.487^b^
≥3∼5	60	58.3			
≥5	21	20.3			
ALT(U/L)	41.19±13.35		32.62±5.39		*p* = 0.000^a^
AST(U/L)	45.24±16.43		31.35±5.5		*p* = 0.000^a^
Platelets(10^9^/L)	134.7±17.61		153.24±22.8		*p* = 0.000^a^

1 BMI: Body mass index, ^2^Occasional: the ethanol intake per week was less than 140 g in men (70 g in women) in the past 12 months.^3^NAS: the NAFLD activity score. ^a^Independent samples-t test. ^b^ Pearson Chi-Square.

### Global analysis of miRNAs by deep sequencing

**Figure 2:**
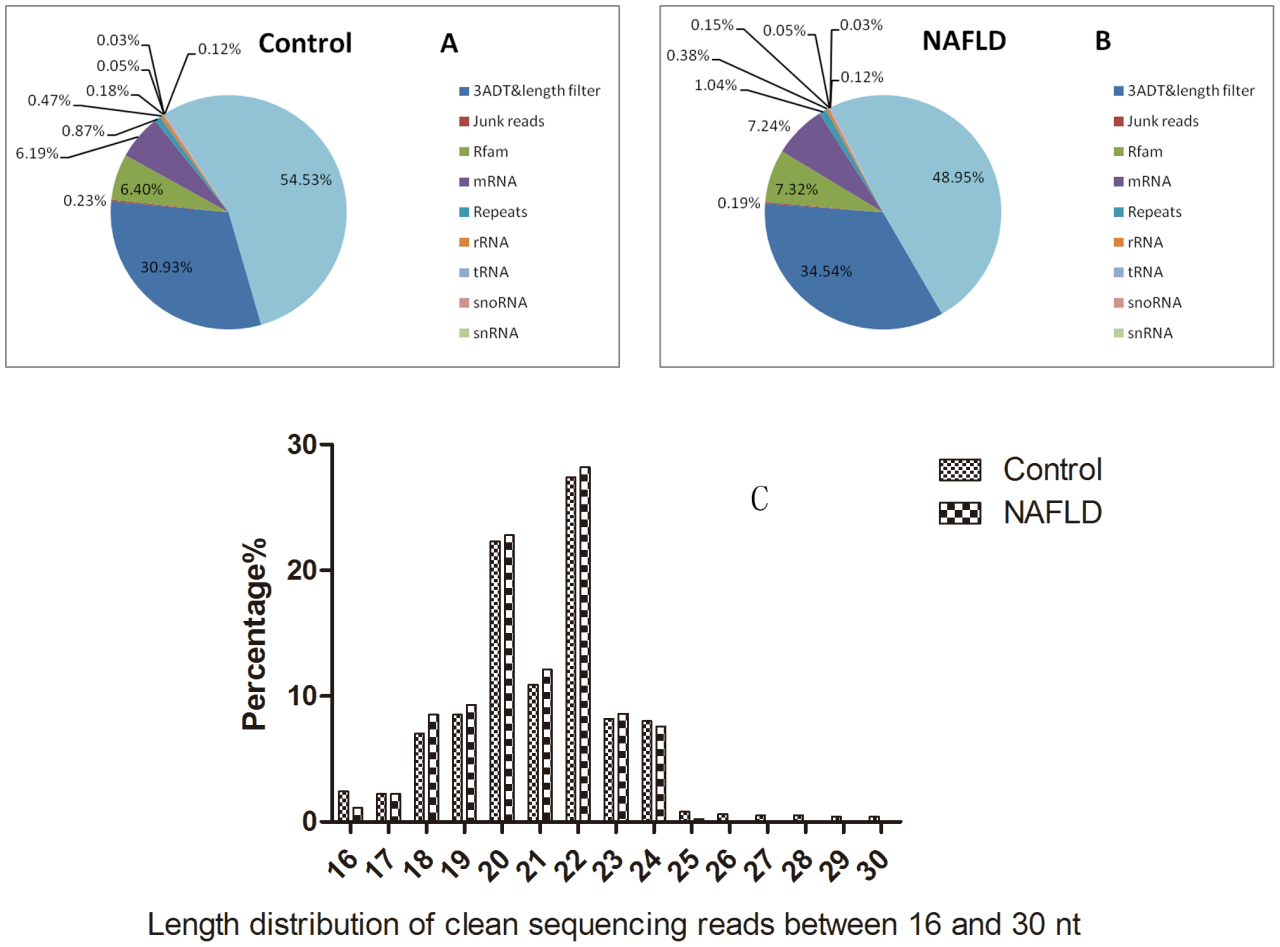
Sequenced reads and distribution of reads. The Illumina GA IIx sequencing of the small RNA library from the serum of healthy controls and NAFLD patients produced 906,910 and 944,362 raw-reads, respectively. After extensive preprocessing and quality control, these raw reads were reduced to 494,523 and 462,263 clean reads, indicating 54.53% and 48.95% of sequenced reads (Figs. 2A, 2B). The entire distribution of reads from 16 to 30 nt is presented in Fig. 2C.

The Illumina GA IIx sequencing of the small RNA library from the serum of healthy controls and NAFLD patients produced 906,910 and 944,362 raw-reads, respectively. After extensive preprocessing and quality control, these raw reads were reduced to 494,523 and 462,263 clean reads, indicating 54.53% and 48.95% of sequenced reads, respectively (Figs. 2A,2B, Table S2). The distribution of all reads from 16 to 30 nt is presented in Fig. 2C. In our study, we found that the length of miRNAs was concentrated on 18 and 24 nt. The clean reads were then mapped to human miRNA (miRs) database v20.0 (ftp://mirbase.org/pub/mirbase/CURRENT/), pre-miRNA (mirs) database v20.0 (ftp://mirbase.org/pub/mirbase/CURRENT/), and genome database (ftp.ncbi.nih.gov/genomes/H sapiens/Assembled chromosomes/seq/). A total of 1,767 unique reads can be mapped to human miRNAs or pre-miRNAs in miRbase, and the pre-miRNAs can be further mapped to the human genome and expressed sequence tag.

### miRNA differential expression profile

The differential expression of miRNA count data was normalized and the number of individual miRNAs reads was standardized by the total number of 1,000,000 reads in each sample. The differential expression levels of 143 miRNAs in the two groups were found to have significant differences. Of these, 6 miRNAs were up-regulated (fold change >2, *P*<0.01) in NAFLD, including hsa-miR-122-5p, hsa-miR-1290, hsa-miR-27b-3p, hsa-miR-192-5p, hsa-miR-148a-3p, and hsa-miR-99a-5p (Table 4).

**Table 4:** Differentially expressed miRNAs between CTL and NAFLD.

no.	miR_name	fold change	up/down	Sequence (5′ to 3′)
1	hsa-miR-122-5p	9.27	up	UGGAGUGUGACAAUGGUGUUUG
2	hsa-miR-1290	4.05	up	UGGAUUUUUGGAUCAGGGA
3	hsa-miR-27b-3p	2.71	up	UUCACAGUGGCUAAGUUCUGC
4	hsa-miR-192-5p	2.61	up	CUGACCUAUGAAUUGACAGCC
5	hsa-miR-148a-3p	2.38	up	UCAGUGCACUACAGAACUUUGU
6	hsa-miR-99a-5p	2.04	up	AACCCGUAGAUCCGAUCUUGUG

### MiRNA expression profile for NAFLD versus control in the training data set

**Figure 3:**
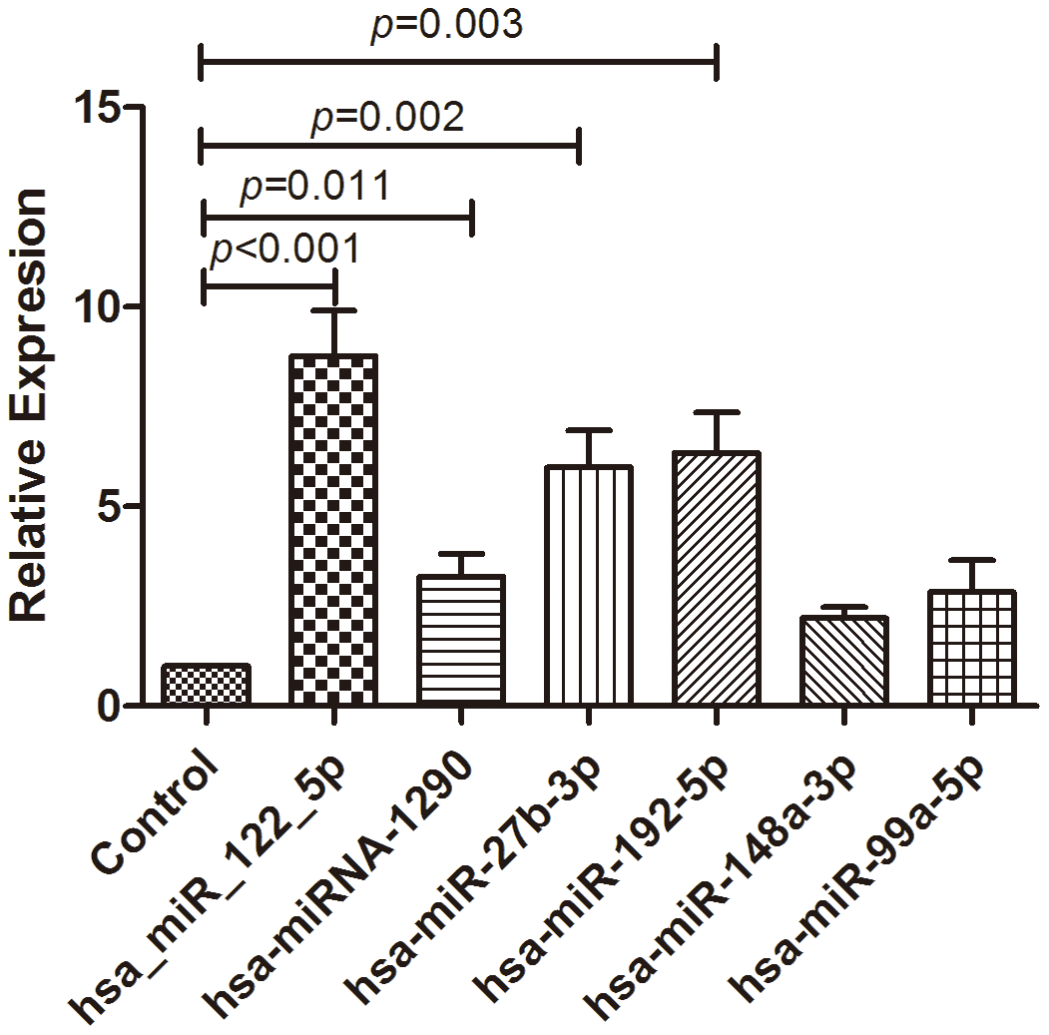
Relative expression of miRNAs between controls and NAFLD patients. Relative expression of 6 candidate miRNAs between controls and NAFLD patients in the training set.

**Figure 4:**
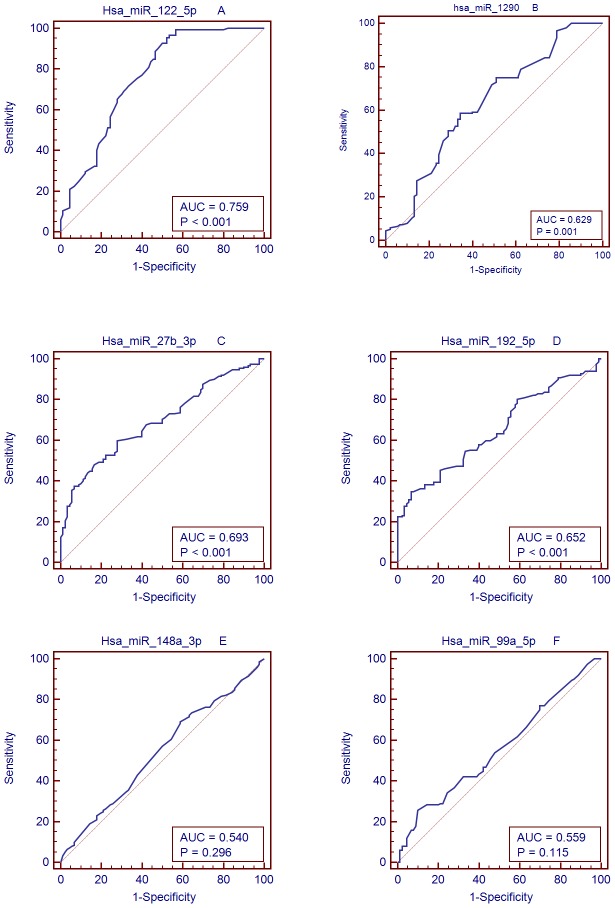
AUC of miRNAs between controls and NAFLD patients. Area under the curve (AUC) of miRNAs. A: miRNA-122; B: miRNA-1290; C: miRNA-27b-3p; D: miRNA-192-5p; E: miRNA-148a-3p; and F: miRNA-99a-5p.

We used qRT-PCR assay to confirm the expression of 6 candidate miRNAs that were selected from the previous step. We identified 4 miRNAs that showed differential expressions, which were selected for the next validation. In the training set, 152 NAFLD patients and 90 controls were examined by qRT-PCR. This phase generated a list of 4 miRNAs that had a significant differential expression pattern (Fig. 3). They were has-miR-122-5p, has-miR-1290, has-miR-27b-3p, has-miR-192-5p.hsa-miR-99a-5p, and has-miR-148a-3p. Compared to Ct of their levels in the control samples. The diagnostic accuracy of these miRNAs, as measured by AUC, was 0.729, 0.629, 0.693, 0.652, 0.54, and 0.559, respectively (Table 5, Fig.4 A–F).

**Table 5:** MicroRNA profile and diagnostic performance in training dataset.

MiRNA group	Sensitivity	Specificity	Ct of criterion	Youden's index J	AUC	p	95% CI
hsa-miR-122-5p	93.4	46.7	≦21.39	0.4338	0.729	<0.001	0.693∼0.862
hsa-miR-1290	58.6	65.6	≦26.85	0.2411	0.629	0.0007	0.555∼0.704
hsa-miR-27b-3p	59.9	72.7	≦22.15	0.3209	0.693	<0.001	0.627∼0.758
hsa-miR-192-5p	34.9	93.3	≦21.23	0.282	0.652	<0.001	0.583∼0.27
hsa-miR-148a-3p	69.1	41.1	≦28.46	0.1019	0.54	0.2961	0.465∼0.615
hsa-miR-99a-5p	25.7	90	≦31.26	0.1566	0.559	0.1154	0.485∼0.633

AUC: area under the receiver operating characteristic curve.

### Establishing the predictive miRNA panel

**Figure 5:**
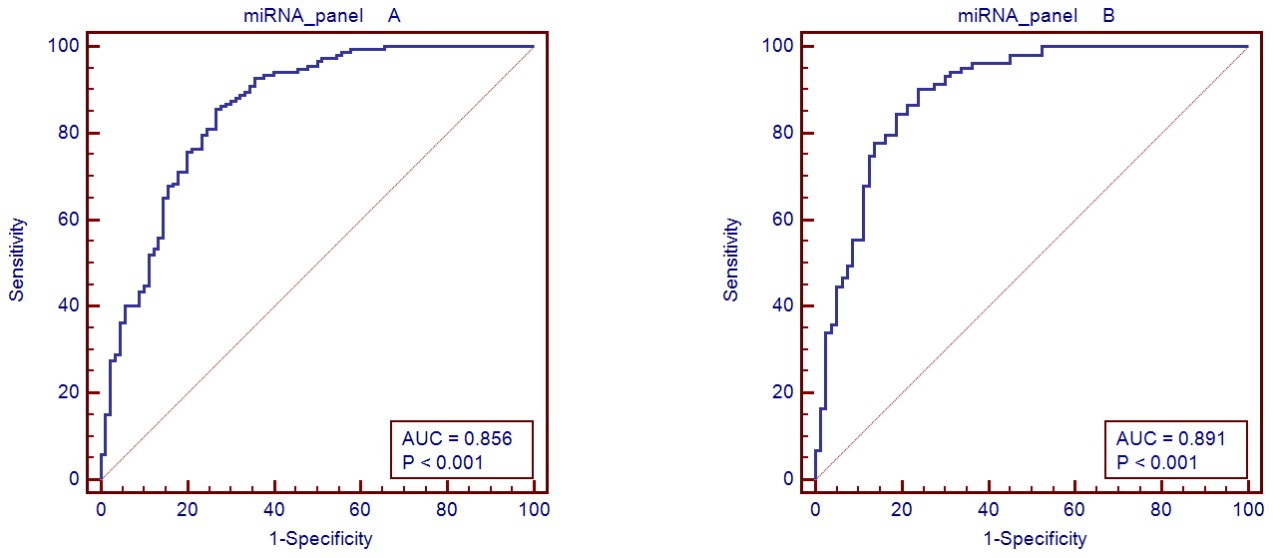
AUC of miRNA panel in the training set and validation set. A: AUC for the miRNA panel in the training set and B: AUC of the miRNA panel in the validation set.

A stepwise logistic regression model to estimate the risk of being diagnosed with NAFLD was applied on the training data set (242 serum samples). All of the four miRNAs turned out to be significant predictors (Table 5). The predicted probability of being diagnosed with NAFLD from the logit model based on the four miRNA panel (Table 6), Logit (P) = 43.9507 -0.91756 miR_122 -0.50132 miR_1290 -0.30842 miR_192 -0.19964 miR_27b was used to construct the ROC curve. The diagnostic performance for the established miRNA panel was evaluated using ROC analysis. The AUC for the miRNA panel was 0.856 (95% CI = 0.804–0.907; sensitivity = 85.55%, specificity = 73.3%, Fig. 5A).

**Table 6:** Logistic regression of miRNAs and miRNAs panel in training dataset.

Variable	Coefficient	Std. Error	Odds ratio	95% CI	P
hsa_miR_122_5p	−0.91756	0.13979	0.3995	0.3038 to 0.5254	<0.0001
hsa_miR_1290	−0.50132	0.13728	0.6057	0.4628 to 0.7927	0.0003
hsa_miR_192_5p	−0.30842	0.11475	0.7346	0.5866 to 0.9199	0.0072
hsa_miR_27b_3p	−0.19964	0.09962	0.819	0.6738 to 0.9956	0.0451
Constant	43.9507				

Enter variable if P<0.05, remove variable if P>0.1; miR_148a_3p and miR_99a_5p not included in the model; Overall model fit: Null model −2 Log Likelihood  = 319.420; Full model −2 Log Likelihood  = 215.317; *x* ^2^ = 104.103, *P*<0.0001.

Logit(P) = 43.9507−0.91756miR_122−0.50132miR_1290−0.30842miR_192−0.19964miR27b; AUC = 0.856.

### Validating the miRNA panel

The parameters estimated from the training data set were used to predict the probability of being diagnosed with NAFLD for the independent validation data set (183 serum samples). Similarly, the predicted probability was used to construct the ROC curve. The AUC of the miRNA panel was 0.891 (95% CI = 0.842–0.941; sensitivity = 90.3%, specificity = 76.2%, Fig. 5B).

**Figure 6:**
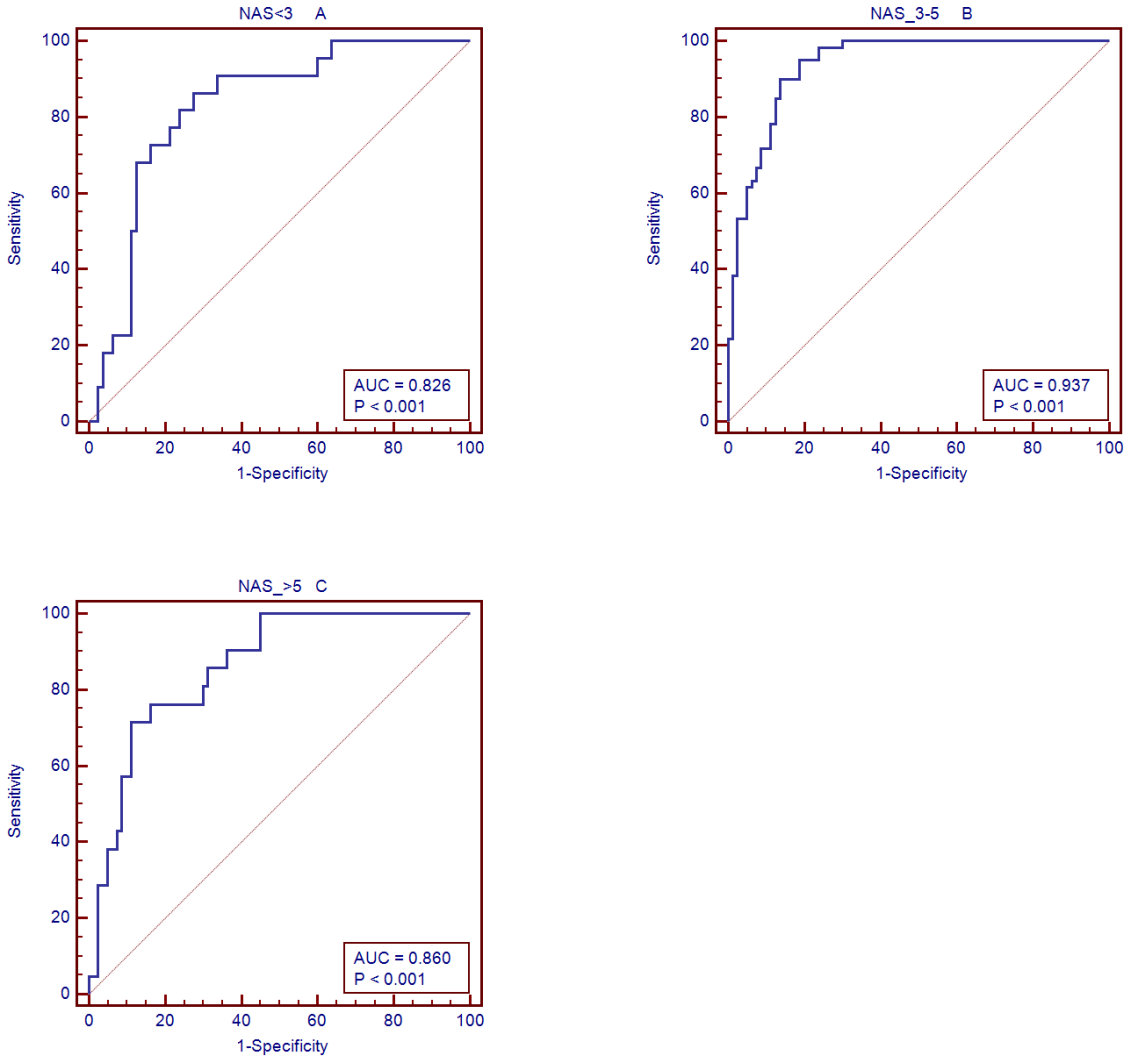
AUC of the miRNA panel in different NAS stages. The corresponding AUCs for patients with NAS stages <3 (A), ≥3 <5 (B), and ≥5 (C) were 0.826, 0.937, and 0.860, respectively.

The diagnostic performance of the miRNA panel in different NAS stages was further evaluated (Figs.6A–C). The corresponding AUCs for patients with NAS stages <3, ≥3, <5, and ≥5 were 0.826, 0.937, and 0.860, respectively. This indicated that the diagnostic performance of the miRNA panel was independent of the disease status, making it an optimal diagnostic tool.

**Figure 7:**
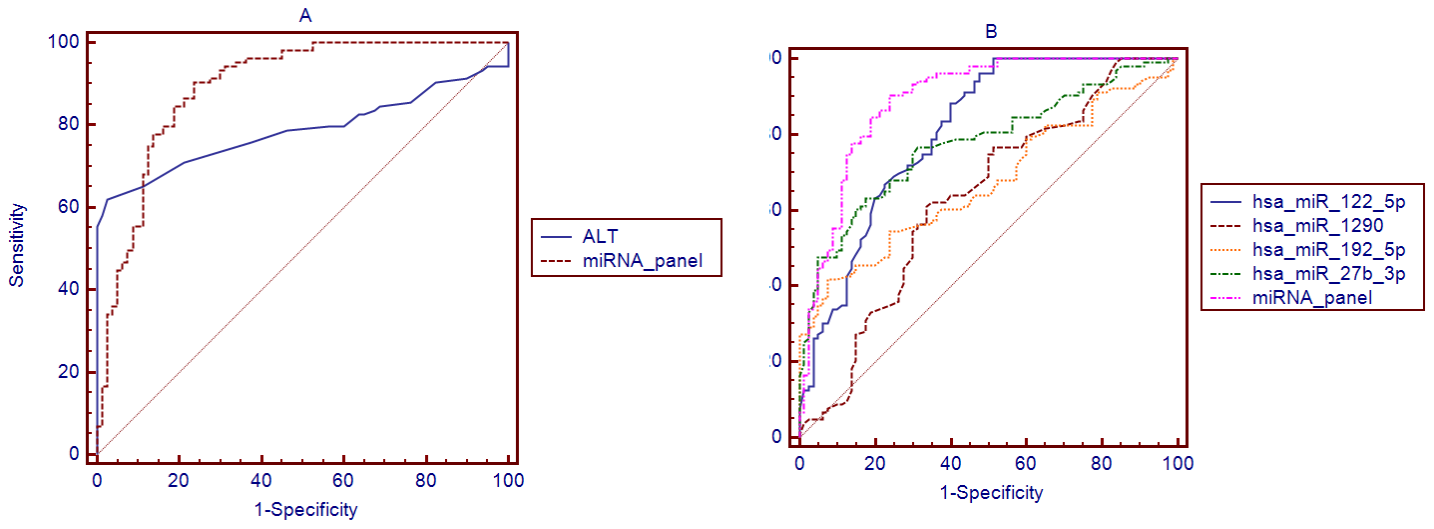
Comparison curves of ROC. A: Comparison curves of ROC between ALT and miRNA panel in the validation set; B: Comparison curves of ROC between each miRNA and miRNA panel in the validation set.

Using the same serum samples, we compared the AUC of the miRNA panel with that of ALT. There was significant difference between the AUC values of the miRNA panel and those of ALT (AUC = 0.786, 95% CI = 0.717–0.855, *P* = 0.142) (Fig. 7A, Table S3). The results indicate that the miRNA panel is a more sensitive and specific biomarker than ALT for NAFLD. We also compared the AUC of the miRNA panel with that of individual miRNA (Fig. 7B, Table S4). There was significant difference between the AUC values of the miRNA panel and individual miRNAs. The results indicate that the miRNA panel has a higher sensitivity and specificity for NAFLD than has-miR-122-5p, has-miR-1290, has-miR-27b-3p, and has-miR-192-5p.

### Compared the AUC of the miRNA panel with that of FIB-4

**Figure 8:**
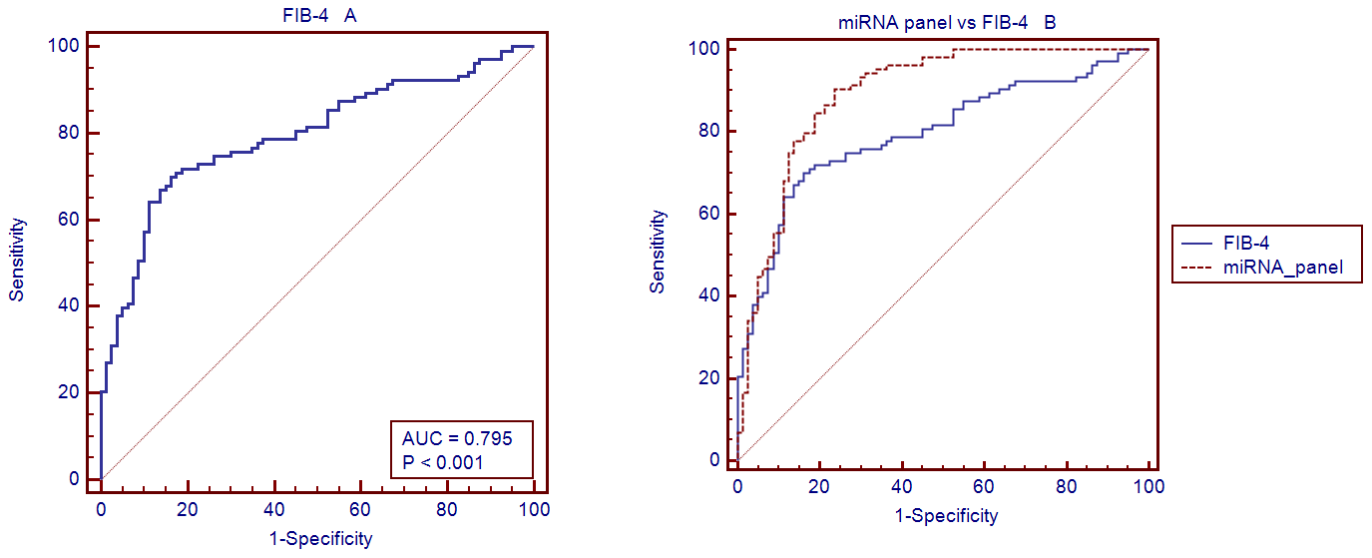
AUC of the FIB-4 and compared with miRNA panel. A: AUC for the FIB-4 in the validation set and B: Comparison curves of ROC with miRNA panel.

FIB-4 is the formula which consists four factors, age, AST, ALT, and platelets in order to pick up NAFLD, FIB-4 = [age(years)×AST(U/L)]/[PLT(10^9^/L)×(ALT U/L)^1/2^]. Using the validation set samples, The diagnostic performance of the FIB-4 was further evaluated (AUC = 0.795, 95% CI = 0.730–0.860; sensitivity = 69.9%, specificity = 83.7%, Fig. 8A). we compared the AUC of the miRNA panel with that of FIB-4. There was significant difference between the AUC values of the miRNA panel and those of FIB-4 (Difference between areas = 0.0962, 95% CI = 0.0152–0.177, *P* = 0.0199) (Fig. 8B). The results indicate that the miRNA panel has a higher sensitivity and specificity for NAFLD than FIB-4.

## Discussion

The past decade has witnessed increasing interest in alternative novel noninvasive strategies for evaluation of NAFLD [21]. These techniques rely on two different but complementary approaches that involve measurement of serum biomarker levels or the use of imaging techniques including conventional ultrasound, CT, MRI, and ultrasound-based elastography for measuring liver stiffness [2]. Several diagnostic panels have been proposed to predict steatosis. SteatoTest [22] incorporates 12 variables in an undisclosed formula, including alpha-2-macroglobulin, haptoglobin, and apolipoprotein A1 [20]. Bedogni et al first proposed Fatty Liver Index (FLI) in 2006, as an algorithm derived from the population of the Dionysos nutrition and liver study [23]. In a recent study, the NAFLD liver fat score was derived from a Finnish population [24]. The score incorporates simple variables such as the presence of the metabolic syndrome and T2DM, fasting serum insulin, aspartate aminotransferase (AST) level, and AST/alanine aminotransferase (ALT) ratio. These serum models have their advantages and disadvantages; however, a common characteristic is that they are composed of known serum biomarkers.

miRNAs are good biomarkers because they are well defined, chemically uniform, restricted to a manageable number of species, and stable in cells and circulation [3]. They could be of diagnostic significance for many liver diseases; however, current literature has been focused on tumors in the liver [25], [26], [27]. Our study revealed that serum miR-122-5p, miR-1290, miR-27b-3p, and miR-192-5p were potential circulating markers for diagnosing NAFLD. The miRNA panel with the four miRNAs from the multivariate logistic regression model demonstrated high accuracy in the diagnosis of NAFLD.

A number of miRNAs are abundantly expressed in the liver; however, miR-122 is liver specific, is estimated to make up 70% of the total hepatic miR complement, and is expressed at high levels [28]. Therefore, miRNA-122 has been the first trial miRNA for miRNA therapeutics since 2008 [29]. In a study by Cermelli et al, NAFLD patients were found to have increased levels of circulating miRNAs such as miR-34a and miR-122 [8]. Inhibition of miR-122 expression in mice leads to down-regulation of cholesterol- and lipid-metabolizing enzymes [30]. miR-122 is known to regulate metabolic pathways in the liver, including cholesterol biosynthesis [31], [32]. Circulating miR-122 levels have been reported to correlate with liver histological stage, inflammation grades, and ALT activity [28], [29], [30], [32], [33].

The present study reported similar results for miR-122 in NAFLD patients, suggesting that the increase in circulating levels of miR-122 is common to chronic liver disease of all etiologies. Previous studies have shown that miR-27 (miR-27a and miR-27b) may play a key role in the progression of atherosclerosis [34]. Ji et al demonstrated that overexpressed miRNA-27a and 27b influence fat accumulation and cell proliferation during the activation of rat hepatic stellate cells [35]. Another study revealed that overexpression of the HCV protein core and NS4B independently activates miR-27 expression. Further, it was established that miR-27 overexpression in hepatocytes results in larger and more abundant lipid droplets. miR-27 expression is thus a novel mechanism contributing to the development of hepatic steatosis [36]. Along with the current study, Cheung et al's study also detected the overexpression of miR-27b in NAFLD patients [11]. MiR-192 is related to cancers such as colon cancer, breast cancer, and gastric carcinoma [37]. Geng et al showed that the expression of miRNA-192 is inversely correlated with the metastatic potential of colon cancer cells [38]. Overexpression of miRNA-192 was found to inhibit metastatic colonization to the liver in an orthotopic mouse model of colon cancer. MiRNA-1290 is also associated with cancers such as laryngeal squamous cell carcinoma, cervical cancer, and pancreatic cancer [39]. Although miR-192 and miRNA-1290 have been found as oncogenes in some cancers, to our knowledge, our study is the first to report the importance of the miR-192 expression profile along with miR-1290 in association with NAFLD.

The first study on the dysregulated miRNA expression pattern in NAFLD was reported by Cheung et al [11]. Out of a total of 46 miRNAs, 23 were underexpressed and 23 were overexpressed; however, they did not have further validation. Other studies on serum miRNA-based disease biomarkers generally focused on individual specific miRNAs [6], [40], [41], [42]. However, the specificity of biomarkers based on a single disease-specific miRNA is generally poor. For example, elevated plasma or serum level of miR-122, which is liver-specific, could result not only from liver cancer but also from HBV infection, cirrhosis, and general liver injury.

Compared with other studies on circulating miRNAs in diagnosing NAFLD, our study is unique for the reasons specified below. First, we screened a large number of serum miRNAs via deep sequencing, which enabled us to better identify potential diagnostic markers. Further, we established an miRNA-panel to diagnose NAFLD and revalidated the panel in a large number of serum samples.Moreover, we compared the AUC of the miRNA panel with those of ALT,miRNA-122 and FIB-4,the miRNA panel is superior to seven other non-invasive markers in NAFLD Patients.

In summary, we identified a serum miRNA panel that differentiates NAFLD patients from healthy controls with a high degree of accuracy in a large number of participants. Our study demonstrates that this serum miRNA panel has considerable clinical value for the diagnosis of NAFLD.

## Supporting Information

Figure S1 **A flow-chart outline of study procedures.**
(TIF)

Table S1 **NAFLD activity score of NAFLD patients in three sets.**
(DOCX)

Table S2 **Overview of reads from raw data to cleaned sequences.**
(DOCX)

Table S3 **Comparison of ROC curves between miRNA panel and ALT in validation set.**
(DOCX)

Table S4 **Comparison of ROC curves between miRNA panel and miRNAs in validation set.**
(DOCX)

## References

[1] Fan JG, Jia JD, Li YM, Wang BY, Lu LG, et al (2011) Guidelines for the diagnosis and management of nonalcoholic fatty liver disease: update 2010: (published in Chinese on Chinese Journal of Hepatology 2010; 18:163–166). J Dig Dis 12: 38–44. 21276207 10.1111/j.1751-2980.2010.00476.x

[2] Castera L, Vilgrain V, Angulo P (2013) Noninvasive evaluation of NAFLD. Nat Rev Gastroenterol Hepatol 10: 666–675. 24061203 10.1038/nrgastro.2013.175

[3] Giordano S, Columbano A (2013) MicroRNAs: new tools for diagnosis, prognosis, and therapy in hepatocellular carcinoma? Hepatology 57: 840–847. 23081718 10.1002/hep.26095

[4] Bala S, Petrasek J, Mundkur S, Catalano D, Levin I, et al (2012) Circulating microRNAs in exosomes indicate hepatocyte injury and inflammation in alcoholic, drug-induced, and inflammatory liver diseases. Hepatology 56: 1946–1957. 22684891 10.1002/hep.25873 PMC3486954

[5] Blanco-Calvo M, Calvo L, Figueroa A, Haz-Conde M, Anton-Aparicio L, et al (2012) Circulating microRNAs: molecular microsensors in gastrointestinal cancer. Sensors (Basel) 12: 9349–9362. 23012546 10.3390/s120709349 PMC3444104

[6] Ge Y, Chen G, Sun L, Liu F (2011) [MicroRNA-29 and fibrosis diseases]. Zhong Nan Da Xue Xue Bao Yi Xue Ban 36: 908–912. 21946200 10.3969/j.issn.1672-7347.2011.09.017

[7] He Y, Huang C, Zhang SP, Sun X, Long XR, et al (2012) The potential of microRNAs in liver fibrosis. Cell Signal 24: 2268–2272. 22884954 10.1016/j.cellsig.2012.07.023

[8] Cermelli S, Ruggieri A, Marrero JA, Ioannou GN, Beretta L (2011) Circulating microRNAs in patients with chronic hepatitis C and non-alcoholic fatty liver disease. PLoS One 6: e23937. 21886843 10.1371/journal.pone.0023937 PMC3160337

[9] Chen YP, Jin X, Xiang Z, Chen SH, Li YM (2013) Circulating MicroRNAs as potential biomarkers for alcoholic steatohepatitis. Liver Int 33: 1257–1265. 23682678 10.1111/liv.12196

[10] Chen YJ, Zhu JM, Wu H, Fan J, Zhou J, et al (2013) Circulating microRNAs as a Fingerprint for Liver Cirrhosis. PLoS One 8: e66577. 23805240 10.1371/journal.pone.0066577 PMC3689750

[11] Cheung O, Puri P, Eicken C, Contos MJ, Mirshahi F, et al (2008) Nonalcoholic steatohepatitis is associated with altered hepatic MicroRNA expression. Hepatology 48: 1810–1820. 19030170 10.1002/hep.22569 PMC2717729

[12] De Minicis S, Day C, Svegliati-Baroni G (2013) From NAFLD to NASH and HCC: pathogenetic mechanisms and therapeutic insights. Curr Pharm Des 19: 5239–5249. 23394093

[13] Fernandez-Hernando C, Ramirez CM, Goedeke L, Suarez Y (2013) MicroRNAs in metabolic disease. Arterioscler Thromb Vasc Biol 33: 178–185. 23325474 10.1161/ATVBAHA.112.300144 PMC3740757

[14] McDaniel K, Herrera L, Zhou T, Francis H, Han Y, et al (2014) The functional role of microRNAs in alcoholic liver injury. J Cell Mol Med 18: 197–207. 24400890 10.1111/jcmm.12223 PMC3930407

[15] Mottin CC, Moretto M, Padoin AV, Swarowsky AM, Toneto MG, et al (2004) The role of ultrasound in the diagnosis of hepatic steatosis in morbidly obese patients. Obes Surg 14: 635–637. 15186630 10.1381/096089204323093408

[16] Kleiner DE, Brunt EM, Van Natta M, Behling C, Contos MJ, et al (2005) Design and validation of a histological scoring system for nonalcoholic fatty liver disease. Hepatology 41: 1313–1321. 15915461 10.1002/hep.20701

[17] Chen C, Ridzon DA, Broomer AJ, Zhou Z, Lee DH, et al (2005) Real-time quantification of microRNAs by stem-loop RT-PCR. Nucleic Acids Res 33: e179. 16314309 10.1093/nar/gni178 PMC1292995

[18] Peltier HJ, Latham GJ (2008) Normalization of microRNA expression levels in quantitative RT-PCR assays: identification of suitable reference RNA targets in normal and cancerous human solid tissues. RNA 14: 844–852. 18375788 10.1261/rna.939908 PMC2327352

[19] Zhang H, Li QY, Guo ZZ, Guan Y, Du J, et al (2012) Serum levels of microRNAs can specifically predict liver injury of chronic hepatitis B. World J Gastroenterol. 18: 5188–5196. 10.3748/wjg.v18.i37.5188 PMC3468850 23066312

[20] Livak KJ, Schmittgen TD (2001) Analysis of relative gene expression data using real-time quantitative PCR and the 2(-Delta Delta C(T)) Method. Methods 25: 402–408. 11846609 10.1006/meth.2001.1262

[21] Castera L, Pinzani M (2010) Non-invasive assessment of liver fibrosis: are we ready? Lancet 375: 1419–1420. 20417845 10.1016/S0140-6736(09)62195-4

[22] Poynard T, Ratziu V, Naveau S, Thabut D, Charlotte F, et al (2005) The diagnostic value of biomarkers (SteatoTest) for the prediction of liver steatosis. Comp Hepatol 4: 10. 16375767 10.1186/1476-5926-4-10 PMC1327680

[23] Bedogni G, Bellentani S, Miglioli L, Masutti F, Passalacqua M, et al (2006) The Fatty Liver Index: a simple and accurate predictor of hepatic steatosis in the general population. BMC Gastroenterol 6: 33. 17081293 10.1186/1471-230X-6-33 PMC1636651

[24] Kotronen A, Peltonen M, Hakkarainen A, Sevastianova K, Bergholm R, et al (2009) Prediction of non-alcoholic fatty liver disease and liver fat using metabolic and genetic factors. Gastroenterology 137: 865–872. 19524579 10.1053/j.gastro.2009.06.005

[25] Gougelet A, Colnot S (2013) [microRNA: new diagnostic and therapeutic tools in liver disease?]. Med Sci (Paris) 29: 861–867. 24148124 10.1051/medsci/20132910013

[26] Gougelet A, Colnot S (2013) MicroRNA-feedback loop as a key modulator of liver tumorigenesis and inflammation. World J Gastroenterol 19: 440–444. 23382622 10.3748/wjg.v19.i4.440 PMC3558567

[27] Hsu SH, Ghoshal K (2013) MicroRNAs in Liver Health and Disease. Curr Pathobiol Rep 1: 53–62. 23565350 10.1007/s40139-012-0005-4 PMC3616382

[28] Hu J, Xu Y, Hao J, Wang S, Li C, et al (2012) MiR-122 in hepatic function and liver diseases. Protein Cell 3: 364–371. 22610888 10.1007/s13238-012-2036-3 PMC4875471

[29] Wang XW, Heegaard NH, Orum H (2012) MicroRNAs in liver disease. Gastroenterology 142: 1431–1443. 22504185 10.1053/j.gastro.2012.04.007 PMC6311104

[30] Iino I, Kikuchi H, Miyazaki S, Hiramatsu Y, Ohta M, et al (2013) Effect of miR-122 and its target gene cationic amino acid transporter 1 on colorectal liver metastasis. Cancer Sci 104: 624–630. 23373973 10.1111/cas.12122 PMC7657140

[31] Esau C, Davis S, Murray SF, Yu XX, Pandey SK, et al (2006) miR-122 regulation of lipid metabolism revealed by in vivo antisense targeting. Cell Metab 3: 87–98. 16459310 10.1016/j.cmet.2006.01.005

[32] Lewis AP, Jopling CL (2010) Regulation and biological function of the liver-specific miR-122. Biochem Soc Trans 38: 1553–1557. 21118125 10.1042/BST0381553

[33] Zhang Y, Jia Y, Zheng R, Guo Y, Wang Y, et al (2010) Plasma microRNA-122 as a biomarker for viral-, alcohol-, and chemical-related hepatic diseases. Clin Chem 56: 1830–1838. 20930130 10.1373/clinchem.2010.147850

[34] Chen WJ, Yin K, Zhao GJ, Fu YC, Tang CK (2012) The magic and mystery of microRNA-27 in atherosclerosis. Atherosclerosis 222: 314–323. 22307089 10.1016/j.atherosclerosis.2012.01.020

[35] Ji J, Zhang J, Huang G, Qian J, Wang X, et al (2009) Over-expressed microRNA-27a and 27b influence fat accumulation and cell proliferation during rat hepatic stellate cell activation. FEBS Lett 583: 759–766. 19185571 10.1016/j.febslet.2009.01.034

[36] Singaravelu R, Chen R, Lyn RK, Jones DM, O'Hara S, et al (2014) Hepatitis C virus induced up-regulation of microRNA-27: a novel mechanism for hepatic steatosis. Hepatology 59: 98–108. 23897856 10.1002/hep.26634

[37] Chiang Y, Zhou X, Wang Z, Song Y, Liu Z, et al (2012) Expression levels of microRNA-192 and -215 in gastric carcinoma. Pathol Oncol Res 18: 585–591. 22205577 10.1007/s12253-011-9480-x

[38] Geng L, Chaudhuri A, Talmon G, Wisecarver JL, Are C, et al.. (2013) MicroRNA-192 suppresses liver metastasis of colon cancer. Oncogene. 10.1038/onc.2013.478 PMC4016997 24213572

[39] Li A, Yu J, Kim H, Wolfgang CL, Canto MI, et al (2013) Serum miR-1290 as a marker of pancreatic cancer—response. Clin Cancer Res 19: 5252–5253. 23881921 10.1158/1078-0432.CCR-13-1899 PMC3783000

[40] Zheng L, Lv GC, Sheng J, Yang YD (2010) Effect of miRNA-10b in regulating cellular steatosis level by targeting PPAR-alpha expression, a novel mechanism for the pathogenesis of NAFLD. J Gastroenterol Hepatol 25: 156–163. 19780876 10.1111/j.1440-1746.2009.05949.x

[41] Shimamura T, Fujisawa T, Husain SR, Kioi M, Nakajima A, et al (2008) Novel role of IL-13 in fibrosis induced by nonalcoholic steatohepatitis and its amelioration by IL-13R-directed cytotoxin in a rat model. J Immunol 181: 4656–4665. 18802068 10.4049/jimmunol.181.7.4656

[42] Cazanave SC, Mott JL, Elmi NA, Bronk SF, Masuoka HC, et al (2011) A role for miR-296 in the regulation of lipoapoptosis by targeting PUMA. J Lipid Res 52: 1517–1525. 21633093 10.1194/jlr.M014654 PMC3137017

