# Supplementary figures and images for: A Pilot Study of Serum MicroRNAs Panel as Potential Biomarkers for Diagnosis of Nonalcoholic Fatty Liver Disease
Source: PLoS One. 2014 Aug 20;9(8):e105192. doi: 10.1371/journal.pone.0105192 (PMC4139327; doi:10.1371/journal.pone.0105192)

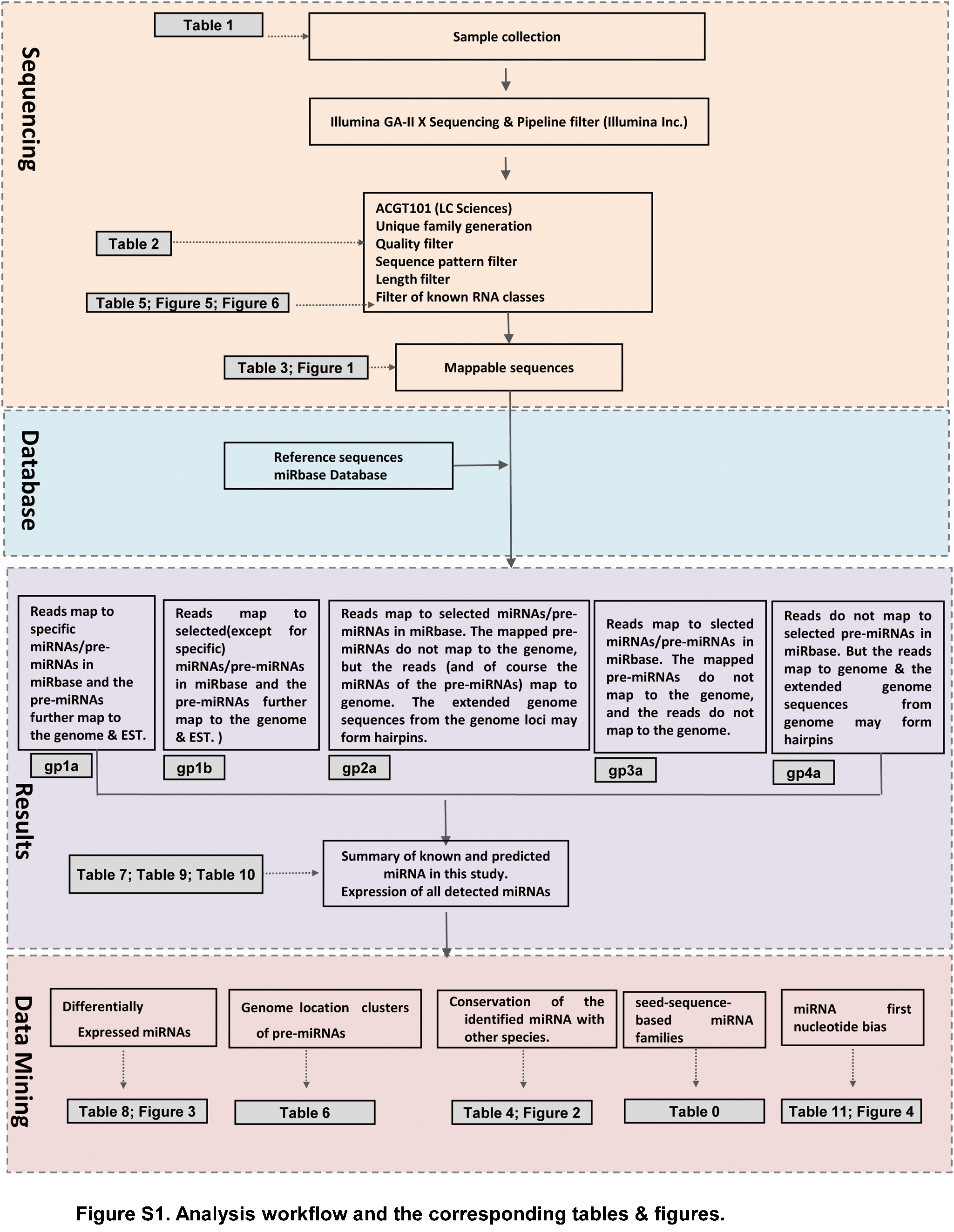

Supplement: Figure S1 — A flow-chart outline of study procedures. (TIF) [file pone.0105192.s001.tif]
