# Supplementary material for: A Pilot Study of Serum MicroRNAs Panel as Potential Biomarkers for Diagnosis of Nonalcoholic Fatty Liver Disease
Source: PLoS One. 2014 Aug 20;9(8):e105192. doi: 10.1371/journal.pone.0105192 (PMC4139327; doi:10.1371/journal.pone.0105192)
Supplement: Table S1 — NAFLD activity score of NAFLD patients in three sets. (DOCX) [file pone.0105192.s002.docx]

Table S1 NAFLD activity score of NAFLD patients in three sets

|  | | | | | | | |
| --- | --- | --- | --- | --- | --- | --- | --- |
| score | screening set(20) | | training set(152) | | validation set(103) | | *p* |
|  | n | % | n | % | n | % |  |
| steatosis |  |  |  |  |  |  |  |
| 0 | 3 | 15 | 21 | 13.81 | 20 | 19.42 | 0.416 |
| 1 | 6 | 30 | 47 | 30.92 | 36 | 23.68 |  |
| 2 | 6 | 30 | 43 | 28.29 | 32 | 30.07 |  |
| 3 | 5 | 25 | 41 | 26.97 | 15 | 16.53 |  |
| lobular inflammation |  |  |  |  |  |  |  |
| 0 | 3 | 15 | 19 | 12.5 | 16 | 15.53 | 0.923 |
| 1 | 13 | 65 | 95 | 62.5 | 69 | 63.1 |  |
| 2 | 4 | 20 | 38 | 25 | 22 | 21.4 |  |
| hepatocellular ballooning |  |  |  |  |  |  |  |
| 0 | 6 | 30 | 45 | 29.61 | 34 | 33.01 | 0.227 |
| 1 | 8 | 40 | 63 | 41.45 | 52 | 50.49 |  |
| 2 | 6 | 30 | 44 | 28.94 | 17 | 16.5 |  |
| Pearson Chi-Square |  |  |  |  |  |  |  |
