# Supplementary material for: A Pilot Study of Serum MicroRNAs Panel as Potential Biomarkers for Diagnosis of Nonalcoholic Fatty Liver Disease
Source: PLoS One. 2014 Aug 20;9(8):e105192. doi: 10.1371/journal.pone.0105192 (PMC4139327; doi:10.1371/journal.pone.0105192)
Supplement: Table S2 — Overview of reads from raw data to cleaned sequences. (DOCX) [file pone.0105192.s003.docx]

Table S2 Overview of reads from raw data to cleaned sequences

|  | | | | | | | | | |
| --- | --- | --- | --- | --- | --- | --- | --- | --- | --- |
|  |  | control | | | | NAFLD | | | |
| type | lib | Total | % of Total | uniq | % of uniq | Total | % of Total | uniq | % of uniq |
| NA | Raw reads | 7896608 | 100 | 906910 | 100 | 9371001 | 100 | 944362 | 100 |
| Sequence type | 3ADT&length filter | 600175 | 7.6 | 280507 | 30.93 | 1309765 | 13.98 | 326183 | 34.54 |
| Sequence type | Junk reads | 4244 | 0.05 | 2097 | 0.23 | 3902 | 0.04 | 1792 | 0.19 |
| RNA class | Rfam | 451536 | 5.72 | 67072 | 7.4 | 524967 | 5.6 | 69142 | 7.32 |
| RNA class | mRNA | 255154 | 3.23 | 56120 | 6.19 | 333047 | 3.55 | 68354 | 7.24 |
| RNA class | Repeats | 81965 | 1.04 | 7919 | 0.87 | 105632 | 1.13 | 9828 | 1.04 |
| RNA class | rRNA | 203358 | 2.58 | 36844 | 0.47 | 207976 | 2.22 | 35413 | 0.38 |
| RNA class | tRNA | 140149 | 1.77 | 13985 | 0.18 | 177572 | 1.89 | 14467 | 0.15 |
| RNA class | snoRNA | 18667 | 0.24 | 3936 | 0.05 | 20524 | 0.22 | 4550 | 0.05 |
| RNA class | snRNA | 22867 | 0.29 | 2709 | 0.03 | 25098 | 0.27 | 3244 | 0.03 |
| RNA class | other Rfam RNA | 66495 | 0.84 | 9598 | 0.12 | 93797 | 1 | 11468 | 0.12 |
| Sequence type | Clean reads | 6677304 | 84.56 | 494523 | 54.53 | 7319212 | 78.1 | 462263 | 48.95 |
