# Supplementary material for: A Pilot Study of Serum MicroRNAs Panel as Potential Biomarkers for Diagnosis of Nonalcoholic Fatty Liver Disease
Source: PLoS One. 2014 Aug 20;9(8):e105192. doi: 10.1371/journal.pone.0105192 (PMC4139327; doi:10.1371/journal.pone.0105192)
Supplement: Table S3 — Comparison of ROC curves between miRNA panel and ALT in validation set. (DOCX) [file pone.0105192.s004.docx]

Table S3 Comparison of ROC curves between miRNAs panel and ALT in validation set

|  | | | | |
| --- | --- | --- | --- | --- |
| Variable | AUC | 95%CI | z statistic | p |
| ALT | 0.786 | 0.717～0.855 | 3.425 | 0.0125 |
| miRNA panel | 0.891 | 0.842～0.941 |  |  |
| Pairwise comparison |  |  |  |  |
