# Supplementary material for: A Pilot Study of Serum MicroRNAs Panel as Potential Biomarkers for Diagnosis of Nonalcoholic Fatty Liver Disease
Source: PLoS One. 2014 Aug 20;9(8):e105192. doi: 10.1371/journal.pone.0105192 (PMC4139327; doi:10.1371/journal.pone.0105192)
Supplement: Table S4 — Comparison of ROC curves between miRNA panel and miRNAs in validation set. (DOCX) [file pone.0105192.s005.docx]

Table S4 Comparison of ROC curves between miRNAs panel and miRNAs in validation set

|  | | | | |
| --- | --- | --- | --- | --- |
| Variable | AUC | 95% CI | z statistic | p |
| hsa_miR_122_5p^a^ | 0.808 | 0.744 to 0.873 | 4.027 | 0.0001 |
| hsa_miR_1290^b^ | 0.633 | 0.550 to 0.716 | 6.187 | <0.0001 |
| hsa_miR_192_5p^c^ | 0.672 | 0.595 to 0.749 | 5.287 | <0.0001 |
| hsa_miR_27b_3p^d^ | 0.775 | 0.709 to 0.842 | 3.429 | 0.0006 |
| miRNA_panel | 0.891 | 0.842 to 0.941 |  |  |
| Pairwise comparison, ^a^miRNA_panel & hsa_miR_122_5p; ^b^miRNA_panel & hsa_miR_1290; ^c^miRNA_panel & hsa_miR_192_5p; ^d^miRNA_panel & hsa_miR_27b_3p | | | | |
